# Zinc Deficiency Disturbs Mucin Expression, *O*-Glycosylation and Secretion by Intestinal Goblet Cells

**DOI:** 10.3390/ijms21176149

**Published:** 2020-08-26

**Authors:** Maria Maares, Claudia Keil, Sophia Straubing, Catherine Robbe-Masselot, Hajo Haase

**Affiliations:** 1 Technische Universität Berlin, Chair of Food Chemistry and Toxicology, Straße des 17. Juni 135, 10623 Berlin, Germany; c.keil@tu-berlin.de (C.K.); Sophia.Straubing93@gmx.de (S.S.); Haase@tu-berlin.de (H.H.); 2 Unité de Glycobiologie Structurale et Fonctionnelle, University of Lille, CNRS, UMR8576-UGSF-Unité de Glycobiologie Structurale et Fonctionnelle, F59000 Lille, France; catherine.robbe-masselot@univ-lille.fr; 3 TraceAge-DFG Research Unit on Interactions of Essential Trace Elements in Healthy and Diseased Elderly, Potsdam-Berlin-Jena, Germany

**Keywords:** zinc deficiency, intestinal mucins, *O*-glycosylation, goblet cells, MUC2, MUC5AC, zinc homeostasis, glycosyltransferases, C1GALT1, B3GNT6

## Abstract

Approximately 1 billion people worldwide suffer from zinc deficiency, with severe consequences for their well-being, such as critically impaired intestinal health. In addition to an extreme degeneration of the intestinal epithelium, the intestinal mucus is seriously disturbed in zinc-deficient (ZD) animals. The underlying cellular processes as well as the relevance of zinc for the mucin-producing goblet cells, however, remain unknown. To this end, this study examines the impact of zinc deficiency on the synthesis, production, and secretion of intestinal mucins as well as on the zinc homeostasis of goblet cells using the in vitro goblet cell model HT-29-MTX. Zinc deprivation reduced their cellular zinc content, changed expression of the intestinal zinc transporters *ZIP-4*, *ZIP-5*, and *ZnT1* and increased their zinc absorption ability, outlining the regulatory mechanisms of zinc homeostasis in goblet cells. Synthesis and secretion of mucins were severely disturbed during zinc deficiency, affecting both *MUC2* and *MUC5AC* mRNA expression with ongoing cell differentiation. A lack of zinc perturbed mucin synthesis predominantly on the post-translational level, as ZD cells produced shorter *O*-glycans and the main *O*-glycan pattern was shifted in favor of core-3-based mucins. The expression of glycosyltransferases that determine the formation of core 1-4 *O*-glycans was altered in zinc deficiency. In particular, *B3GNT6* mRNA catalyzing core 3 formation was elevated and *C2GNT1* and *C2GNT3* elongating core 1 were downregulated in ZD cells. These novel insights into the molecular mechanisms impairing intestinal mucus stability during zinc deficiency demonstrate the essentiality of zinc for the formation and maintenance of this physical barrier.

## 1. Introduction

The essential micronutrient zinc is required for catalytic, structural, and regulatory functions of various zinc-metalloproteins in the human body [1]. Accordingly, deprivation of this metal is associated with severe health consequences [2]. Prolonged deficiency enhances the risk of infection, often connected with diarrhea and impaired wound healing, resulting in high morbidity [3]. This micronutrient deficiency affects about 16% of the world’s population [4] and is directly connected with inadequate zinc absorption in the intestinal tract, as zinc has to be replenished in order to counterbalance daily zinc losses [5]. To this end, insufficient zinc intake, nutrition with low zinc bioavailability, as well as diseases associated either with zinc malabsorption or increased zinc losses, such as *acrodermatitis enteropathica*, inflammatory bowel diseases, and diarrhea, can cause zinc deficiency [3].

As tissues with high turnover rates are particularly impaired by zinc deficiency [6], the intestinal tract is severely affected; this is mainly manifested by morphological changes [7,8] and severe degeneration [9] of the intestinal epithelium. The disruption of this barrier in zinc deficiency is further enhanced by a reduction in its integrity, resulting in increased membrane permeability [10]. This is accompanied by reduced self-renewal of the epithelium due to decreased crypt cell proliferation [11] and altered function of epithelial cells, illustrated by impaired activity of brush border enzymes [12]. There is evidence that zinc deficiency also affects the production of the gastrointestinal mucus layer, as reduced amounts of mucus and alteration of its composition were detected in zinc-deficient (ZD) rats and sheep [13,14]. However, the underlying molecular mechanism and cellular processes causing this deterioration remain to be investigated.

The mucus layer is critical for gastrointestinal health and function. It covers the whole gastrointestinal tract (GIT) and serves as an additional physical barrier for the underlying epithelium, protecting it against chemical and physical damage and pathogens [15]. It is also a habitat for a wide range of commensal bacteria in the colon [16], was shown to be essential for intestinal gastrointestinal immunity [16] and is important for nutritional absorption of macro- as well as micronutrients, such as zinc [17]. The main structural component of the mucus layer are mucins, accounting for ~5–10% of this barrier; apart from ~95% water, the remainder is non-mucin proteins, salts, and lipids [15]. These highly glycosylated proteins are relevant for the physicochemical properties and viscoelasticity of the mucus [15] and are produced and secreted by specialized mucin-producing goblet cells [16]. Secreted gel-forming mucins are large network-like structured polymers (approximate molecular mass of intestinal mucins: ~2.5 MDa [18]). These are built from MUC monomers, mainly consisting of proline/threonine/serine tandem repeats, forming the so-called protein backbone, which is extensively covered by *O*-linked oligosaccharides [19]. These *O*-glycans protect the protein backbone against bacterial degradation and are pivotal for the high water-binding capacity and gel-forming properties of mucins when secreted into the intestinal lumen [19]. Alterations of the *O*-glycan pattern, which is highly diverse between individuals [20], are associated with several gastrointestinal diseases [19]. Hence, a disturbance of the barrier during zinc deficiency could have serious consequences for intestinal health and homeostasis.

Even though the degeneration of intestinal mucus in zinc-restricted animals was described about 45 years ago, the effect of this nutrient deficiency on synthesis and secretion of intestinal mucins on the cellular level has not yet been elucidated. Zinc is important for the activity and function of the gastrointestinal tract and its homeostasis was widely investigated in enterocytes and Paneth cells (reviewed in [21]) but not studied in goblet cells, so far. This study aims to illuminate the impact of zinc deficiency on zinc homeostasis, synthesis, and *O*-glycosylation of MUC apo-proteins, as well as on the secretion of intestinal mucins by goblet cells using the HT-29-MTX in vitro model for intestinal mucus-producing goblet cells.

## 2. Results and Discussion

### 2.1. Characterization of Zinc-Deficient Goblet Cells

In order to subject goblet cells to ZD, HT-29-MTX cells were cultured in chelexed medium. Treatment of cell culture medium with Chelex^®^ 100 Resin [22] or other iminodiacetate-containing polymers [23] is a common procedure to remove zinc and to induce zinc deficiency in vitro. However, the incubation of monocytes in chelexed medium affected cellular cytokine production independently from zinc deprivation [22], and an impact on other cellular parameters cannot be excluded. Consequently, in the present, study experiments were also conducted in cells cultivated in chelexed medium replenished with zinc (ZA). No significant differences between cells cultivated with control (CTR) and ZA medium were detected. Accordingly, the results of this study are solely based on zinc restriction.

**Figure 1:**
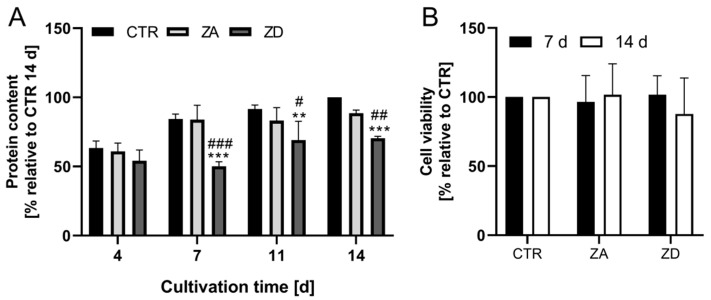
Impact of zinc deprivation on cellular protein and cell viability of goblet cells. HT-29-MTX cells were cultivated for 4–14 days with control (CTR), zinc-adequate (ZA) or zinc-deficient (ZD) medium, respectively. (**A**) The amount of cellular protein was determined by sulforhodamine B (SRB) assay and is shown relative to protein content of cells grown in CTR medium after 14 days. (**B**) Cell viability was determined by measuring dehydrogenase activity using 3-(4,5-Dimethylthiazol-2-yl)-2,5-diphenyltetrazolium bromide (MTT). All data are presented as means + SD of three independent experiments. Significant differences to CTR (** *p* < 0.01; *** *p* < 0.001) and to ZA medium (^#^ *p* < 0.05; ^##^ *p* < 0.01; ^###^ *p* < 0.001) within the same cultivation time are indicated (Two-way analysis of variance (ANOVA) with Bonferroni post hoc test).

Based on experiments by Hennebicq-Reig et al., the different cellular states of HT-29-MTX investigated in the present study are pre-confluent (cultured for 4 days), confluent (7 days) and post-confluent cells (14 days) [24]. Protein of zinc-sufficient HT-29-MTX increased with cultivation time (Figure 1A). In agreement with previous studies with HT-29-MTX clones, cells start to differentiate after reaching confluence, accompanied by the beginning mucus secretion and cell polarization [24,25]. Thus, slightly increased levels of protein from day 7 to 14 might be, at least partly, due to enhanced mucin secretion. The elevation of mucin secretion with progressing differentiation of HT-29-MTX was additionally confirmed by histochemical staining of secreted mucins, showing increased mucus production up to day 14 (Supplementary Figure S1). Cultivating goblet cells under ZD conditions, however, leads to significantly lower cellular protein (Figure 1A) with 40% or 30% less protein in confluent or post-confluent cells, respectively. In contrast, the cell viability of goblet cells was not altered by zinc depletion (Figure 1B). Accordingly, the decline in cellular protein of ZD HT-29-MTX can be associated with retardation in cell growth as well as impaired mucin secretion as a result of zinc restriction, resembling the decrease in mucosal protein and impaired cell proliferation in zinc-restricted animals [11,12].

### 2.2. Zinc Homeostasis of Goblet Cells

**Figure 2:**
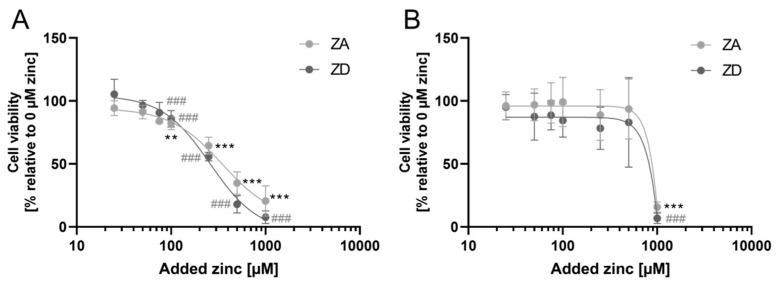
Impact of zinc deficiency on cellular zinc toxicity. HT-29-MTX cells were cultivated for 7 days (**A**) or 14 days (**B**), respectively, in zinc-deficient (ZD) or -adequate (ZA) medium. Cells were treated with different zinc concentrations for 24 h and metabolic activity was measured with MTT. Data are presented as means ± SD of three independent experiments. Significant differences to 0 µM zinc are indicated (** *p* < 0.01; *** *p* < 0.001;^, ###^ *p* < 0.001; one-way ANOVA with Dunnett’s multiple comparison test). Parameters of non-linear regression summarized in Supplementary Table S1.

ZD and zinc-sufficient goblet cells were treated with different zinc concentrations (0–1000 µM) to assess their robustness against zinc toxicity, showing that the cellular zinc status had no significant impact on their survival (Figure 2A,B).

**Figure 3:**
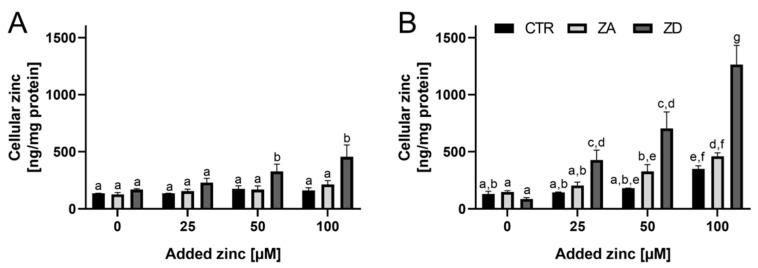
Zinc uptake of HT-29-MTX during zinc deficiency. HT-29-MTX cells were cultured for 7 days (**A**) and 14 days (**B**) in CTR, zinc-adequate (ZA) or -deficient (ZD) medium. Cellular zinc after treatment with 0–100 µM zinc for 24 h was quantified with inductively coupled mass spectrometry (ICP–MS) and is presented relative to protein content of the cells. Data are shown as means + SD of three independent experiments. Bars sharing letters are not significantly different (Two-Way ANOVA with Bonferroni post-hoc test).

To further examine zinc homeostasis in goblet cells during zinc deficiency, cellular uptake of extracellular-added zinc was studied. Zinc content of confluent zinc-sufficient HT-29-MTX cells did not change (Figure 3A). Zinc restriction for 7 days did not impact basal zinc levels of goblet cells, yet confluent ZD cells absorbed significantly higher amounts upon adding 50 and 100 µM zinc than those cultured in CTR and ZA. Zinc deprivation of HT-29-MTX for two weeks significantly reduced basal zinc levels by 42% (Figure 3B; zinc content: CTR 131.4 ± 22.6 ng/mg protein; ZA 148.7 ± 10.0 ng/mg protein; ZD 85.7 ± 12.0 ng/mg protein). In contrast to confluent ZA and CTR cells, zinc uptake by zinc-sufficient post-confluent cells significantly increased their cellular zinc content. ZD post-confluent goblet cells absorbed significantly more zinc, raising their basal zinc level by 8 (50 µM zinc) and 14 (100 µM zinc) times, respectively (Figure 3B).

**Figure 4:**
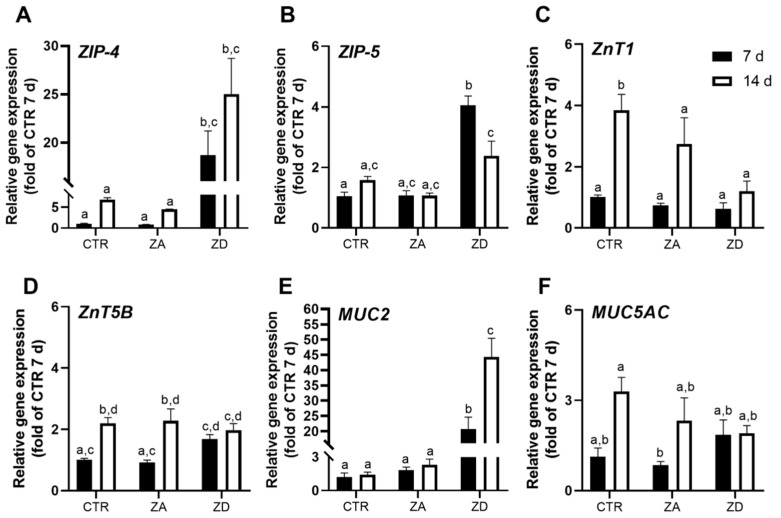
Influence of zinc deficiency on the expression of intestinal zinc transporters (**A**–**D**) and mucins (**E**,**F**). HT-29-MTX were cultured for 7 and 14 days in CTR, zinc-adequate (ZA) or -deficient (ZD) medium, respectively, and gene expression analyzed by quantitative real-time PCR (qPCR). Data are presented as means + standard error of mean (SEM) of three independent experiments. Bars sharing letters are not significantly different (Two-Way ANOVA with Bonferroni post-hoc test).

Next, we analyzed the expression of selected zinc transporters known to mediate intestinal zinc absorption [21]. Zinc homeostasis is mainly regulated by members of two zinc transporting families: zinc transporter (ZnT) and Zrt- and Irt-like protein (ZIP). In the intestinal tract, zinc is absorbed into intestinal epithelial cells via ZIP-4 at their apical membrane and exported into the blood by basolaterally localized ZnT1. ZIP-5 at the basolateral membrane transports systemic zinc from the blood back into the intestinal epithelial cells, whereas the bidirectional transporter ZnT5 variant B (ZnT5B) at the apical membrane is discussed to export cellular zinc into the intestinal lumen as well as import the metal into cells [2]. To date, there are no studies on their expression pattern in HT-29-MTX. The parental intestinal cell line HT-29, however, expresses the main zinc transporters ZIP-4 and ZnT1 [26]. Expression of *ZIP-4*, *ZnT1,* and *ZnT5B* were upregulated in zinc-sufficient goblet cells with ongoing differentiation (Figure 4; C, *ZnT1*: *p* < 0.05 CTR 7 days vs. CTR 14 days; D, *ZnT5B*: *p* < 0.05 CTR 7 days vs. CTR 14 days). Solely the expression of the importer *ZIP-5* did not change in HT-29-MTX of differing maturity (Figure 4B). These alterations indicate that the zinc homeostasis of goblet cells is developing during their differentiation, similar to what is described in human enterocytes [27], which might explain the higher zinc absorption ability of post-confluent HT-29-MTX compared to cells that just reached confluency (Figure 3).

Zinc deprivation of goblet cells severely affected the expression of zinc transporters, significantly increasing *ZIP-4* mRNA in both confluent and post-confluent cells, leading to 18-fold (Figure 4A; 7 days: *p* < 0.001 ZD vs. CTR and *p* < 0.001 ZD vs. ZA) and 25-fold higher (Figure 4A; 14 days: *p* < 0.001 ZD vs. CTR and *p* < 0.001 ZD vs. ZA) *ZIP-4* expression compared to ZA and CTR, respectively. Likewise, *ZIP-5* mRNA significantly increased in confluent ZD cells (Figure 4B; 7 days: *p* < 0.001 ZD vs. CTR and *p* < 0.001 ZD vs. ZA). However, *ZIP-5* levels significantly declined again when cultured for additional 7 days without zinc (Figure 4B; ZD 7 days vs. ZD 14 days: *p* < 0.01). Zinc deficiency did not impair *ZnT-1* mRNA levels in confluent cells, whereas zinc depletion for 14 days significantly decreased *ZnT-1* expression (Figure 4C; CTR 14 days vs. ZD 14 days: *p* < 0.05). Levels of *ZnT5B* mRNA were not affected in post-confluent cells regardless of their zinc status. Deprivation for 7 days, however, upregulated *ZnT5B* to an amount almost similar to its expression in post-confluent cells (Figure 4D).

These results demonstrate that zinc availability critically affects the zinc homeostasis of goblet cells. The zinc requirements of ZD cells are probably elevated. Hence, they might absorb more zinc (Figure 3B) in order to maintain cellular zinc homeostasis, similar to what is known for enterocytes (reviewed in [2]). The differential regulation of intestinal zinc transporters in response to nutritional zinc controls zinc absorption and distribution in vivo [2]. Intestinal ZIP-4 and ZIP-5 are known to be predominantly regulated in a translational and post-translational manner during zinc deficiency [2], yet upregulation of *ZIP-4* mRNA in zinc deficiency was already reported in in vivo small intestine of ZD rats or mice [28,29]. Likewise, it is known that cellular zinc levels regulate ZnT1 expression via metal regulatory transcription factor 1 (MTF1) [30]. During zinc deficiency, membrane-bound ZnT1 is degraded [31] and its mRNA levels were described to decrease in mouse pancreas [29] as well as in the intestine of weaning rats [32]. Accordingly, gene expression changes of these transporters in post-confluent zinc-restricted goblet cells might be another reason for their enhanced zinc uptake (Figure 3B). While the elevated *ZIP-4* and *ZIP-5* mRNA expression may lead to increased zinc uptake, less metal could be exported as the main zinc exporter *ZnT1* is downregulated in ZD goblet cells. Moreover, impaired mucus production and secretion during zinc deficiency might also influence zinc absorption by ZD cells. By comparing short-term zinc uptake of confluent HT-29-MTX cells in the presence of mucins and after the removal of the mucus layer [17], it was recently demonstrated that the absence of mucus enhances zinc uptake by goblet cells. Hence, diminished mucus layer during zinc deficiency could decrease the zinc buffering capacity provided by this extracellular barrier, resulting in higher zinc availability for the underlying cells and increased zinc uptake.

### 2.3. Intestinal Mucin Synthesis and Secretion during Zinc Deficiency

In order to elucidate the underlying mechanisms reducing the intestinal mucus layer during zinc deficiency in vivo, mucin synthesis and secretion were examined in ZD HT-29-MTX. Gene expression of the secreted and gel-forming *MUC2* and *MUC5AC* were determined to evaluate transcriptional changes of mucins during zinc deficiency. While *MUC2* encodes the main small and large intestinal mucin [18], MUC5AC is mainly produced in the human stomach [19]; yet, it is highly expressed in HT-29-MTX [25]. In the presence of zinc *MUC5AC* expression increased with ongoing differentiation, leading to 3.5 times higher levels in post-confluent cells (Figure 4F). *MUC2* levels, on the other hand, did not differ between CTR and ZA cells of varying maturity (Figure 4E). These results reiterate previous reports on MUC regulation in HT-29-MTX [25] and corroborate their elevated mucin secretion during differentiation (Supplementary Figure S1) [24,25].

During zinc deficiency, the expression of mucins is altered. While *MUC5aC* tends to decrease in zinc-depleted cells (Figure 4F), zinc restriction led to significantly higher *MUC2* expression (Figure 4E; 7 days: *p* < 0.01 ZD vs. CTR, *p* < 0.05 ZD vs. ZA; 14 days: *p* < 0.001 ZD vs. CTR, *p* < 0.001 ZD vs. ZA).

**Figure 5:**
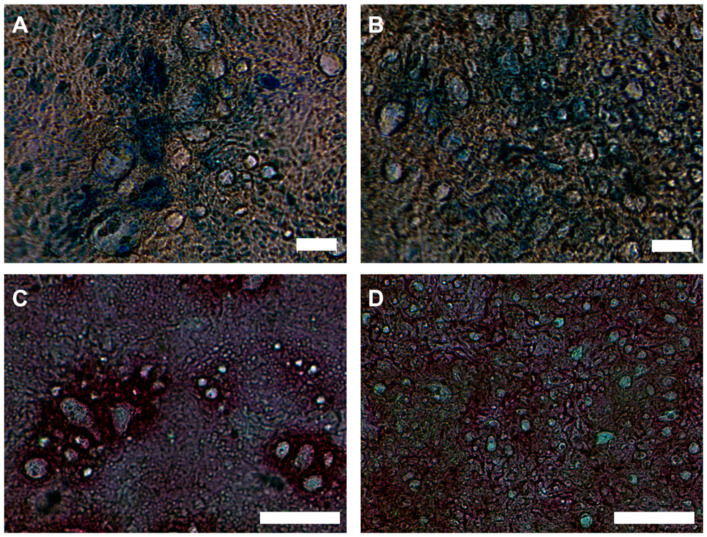
Staining of secreted mucins. CTR (**A**,**C**) and ZD (**B**,**D**) cultivation of HT-29-MTX for 14 days. Histological staining of mucins was performed with alcian blue (**A**,**B**) and PAS (**C**,**D**). Shown are representative images from three independent experiments. Scale bar 50 µm.

Mucus secreted by goblet cells during zinc deficiency was visualized with two histological staining methods, alcian blue (AB), which stains acidic mucins, such as sulfated and sialylated mucins [33], and periodic acid-Schiff (PAS), detecting neutral glycoproteins [34]. Post-confluent ZD and CTR cells were used because MUC expression (Figure 4F) and mucin secretion increased up to day 14 of cell cultivation (Supplementary Figure S1). Microscopic images of zinc-sufficient HT-29-MTX show characteristic cytoplasmic mucin granules and intercellular mucin inclusions, so-called “mucin lakes”, which have been described before in in vitro goblet cells [35]. Intense AB- (Figure 5A) as well as PAS-staining (Figure 5C) mainly in close vicinity of these mucin storages demonstrate overall secretion of acidic as well as neutral mucins by CTR cells. In contrast, mucin staining of ZD HT-29-MTX is less intense (Figure 5B,D), particularly with less PAS-positive glycoproteins (Figure 5D), indicating decreased mucin secretion. Moreover, while the number of mucin granules appears to be increased, they are considerably smaller and show less staining (Figure 5B,D) than in CTR cells (Figure 5A,C). Hence, similar to the diminished mucus barrier described in ZD animals [13,14], the human goblet-cell line HT-29-MTX displays a disturbed mucus layer and impaired mucin secretion upon zinc deprivation.

These findings demonstrate that zinc directly or indirectly influences mucin expression and secretion in goblet cells. Based on zinc supplementation studies in pigs, there is evidence for a direct interrelation of MUC expression and nutritional zinc status [36]. The chronic autosomal recessive disorder cystic fibrosis (CF) is associated with mucus accumulation predominantly in lung and GIT [37] and discussed to be linked to the body’s zinc status [38]. Recently, MUC overexpression in in vitro CF lung epithelial cells was connected with decreased intracellular available zinc levels, generated with the metal chelator *N*,*N*,*N*′,*N*′-tetrakis(2-pyridylmethyl) ethylenediamine (TPEN) [39]. The overexpression of *MUC2* and *MUC5AC* mRNA as well as mucin hypersecretion by intestinal goblet cells frequently occurs during inflammation in the GIT, probably as a first-line defense mechanism of the intestinal epithelium against bacterial infections [16]. The observed *MUC2* upregulation in ZD cells might possibly function as a strategy to counterbalance the disturbed mucus layer during zinc deficiency. The regulatory mechanisms controlling the *MUC* gene expression during zinc deficiency remain unclear, but as zinc deficiency in humans is associated with increased inflammation [40], similar mechanisms might lead to the upregulation of *MUC2* mRNA in zinc-deprived goblet cells.

**Figure 6:**
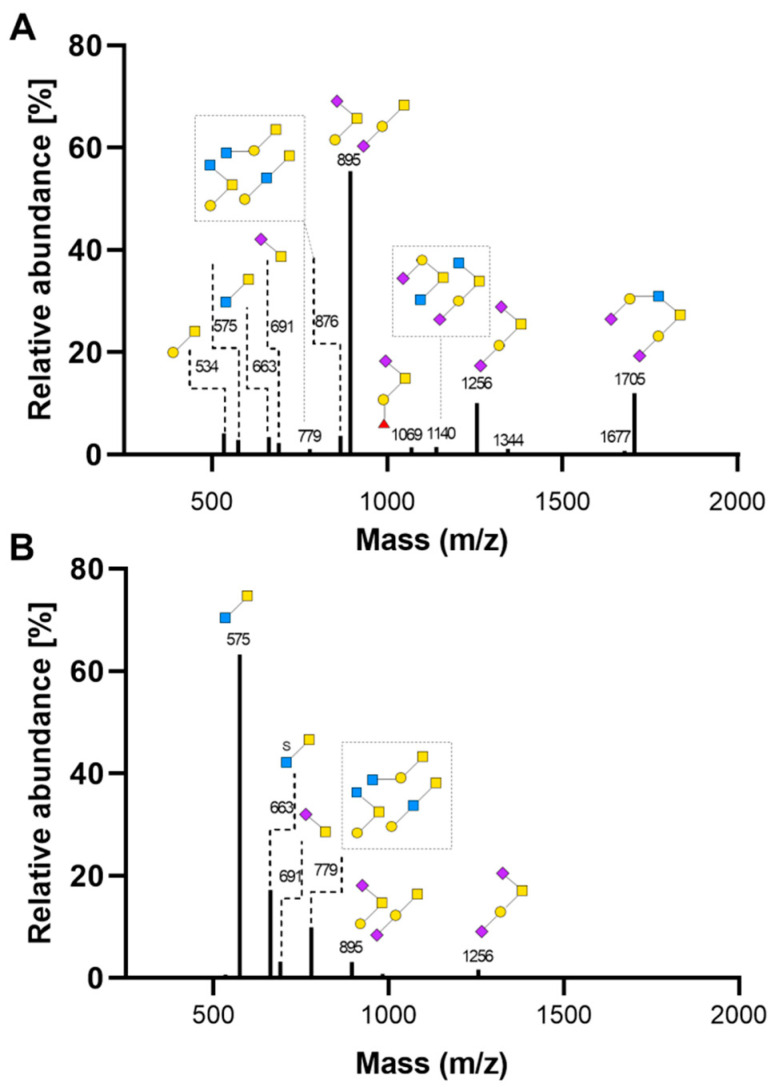
Impact of zinc deficiency on the *O*-glycosylation pattern of mucins secreted by HT-29-MTX. Cells were cultured for 14 days in zinc-sufficient (**A**) or -deficient medium (**B**). The main glycan structures found in supernatants from these cells are depicted according to the symbol nomenclature for glycans (SNFG) [44]. Data are shown as means of three independent experiments.

MUC apo-proteins are extensively co- and post-translationally modified, including *N*-glycosylation and dimerization in the endoplasmic reticulum, followed by *O*-glycosylation in the Golgi [41]. Human *O*-glycan biosynthesis is initiated by the addition of *N*-acetyl-galactosamine (GalNAc) to serine and threonine residues of the MUC protein backbone, mediated by a large family of UDP-GalNAc:polypeptide GalNAc transferases (GALNAC-Ts), forming GalNAc-Ser/Thr (Tn antigen) [41]. The latter is either sialylated by GalNAc α-2, 6-Sialyltransferase 1 (ST6GALNAC1), adding *N*-acetylneuraminic acid (NeuAc), or elongated forming core 1-4 *O*-glycan structures [41]. These core glycans are further elongated with oligosaccharides or terminated by sialyation or fucosylation, resulting in a highly heterogeneous *O*-glycan pattern of mucins [19]. To investigate whether the lack of zinc alters mucin *O*-glycosylation, *O*-glycan pattern of mucins secreted by HT-29-MTX was analyzed by matrix-assisted laser desorption/ionization time-of-flight (MALDI-TOF) (Figure 6; structures of detected *O*-glycans are summarized in Supplementary Table S2). Post-confluent zinc-sufficient HT-29-MTX cells (Figure 6A) mainly secreted mucins based on Thomsen-Friedenreich (TF) antigens (Galβ1-3GalNAc), a core 1 type mucin, similar to a recent study with differentiating HT-29-MTX [42]. A total of 55.4% of the detected *O*-glycans in supernatants of CTR cells were sialylated (NeuAcα2-3Galβ1-3GalNAc or Galβ1-3(NeuAcα2-6)GalNAc; *m*/*z* 895) and 10.1% disialylated TF antigens (NeuAcα2-3Galβ1-3(NeuAcα2-6)GalNAc; *m*/*z* 1256). Besides, 12% long *O*-glycans based on the core 2 type (NeuAcα2-3Galβ1-3(NeuAcα2-3Galβ1-4GlcNAcβ1-6)GalNAc; *m*/*z* 1705) were found. Together with a small percentage of sialylated Tn antigens (NeuAcα2-6GalNAc), 79.8% of glycans produced by this cell line are sialylated, confirming previous findings [42,43]. Secreted mucins are indeed highly acidic, as already demonstrated with AB staining. The remaining *O*-glycans in supernatants of HT-29-MTX were small amounts of shorter *O*-glycans, composed of 4.1% Galβ1-3GalNAc (TF antigen), 2.8% GlcNAcβ1-3GalNAc (core 3) and 3.4% sulfated core 3.

Under ZD conditions, fewer *O*-glycans were detected, which is in line with the decreased overall mucin secretion in zinc deficiency. *O*-glycosylation of secreted mucins was critically affected in zinc-restricted goblet cells, producing significantly higher amounts of short *O*-glycans than in CTR cells (Figure 6A). The sialylation of mucins is particularly disturbed in zinc deficiency, as only 8.1% of detected *O*-glycans were sialylated, corresponding to a reduction in their abundance by more than 70%. Moreover, major *O*-glycans that were found in supernatants of CTR cells are less abundant in the absence of zinc. Zinc-depleted cells secreted 52.3% less sialylated (*m*/*z* 895) and 8.4% less disialylated TF antigens (*m*/*z* 1256). Furthermore, the long core-2-based *O*-glycan at *m*/*z* 1705 was not detected at all. In contrast, major mucins found after zinc deprivation are based on the core 3 glycan GlcNAcβ1-3GalNAc (*m*/*z* 575) and its sulfated form (*m*/*z* 663), representing 80.5% of *O*-glycans secreted by ZD cells. Apart from these two glycans, only the sialyl-Tn antigen was found in ZD cell supernatants, with comparable amounts being detected in CTR HT-29-MTX.

**Figure 7:**
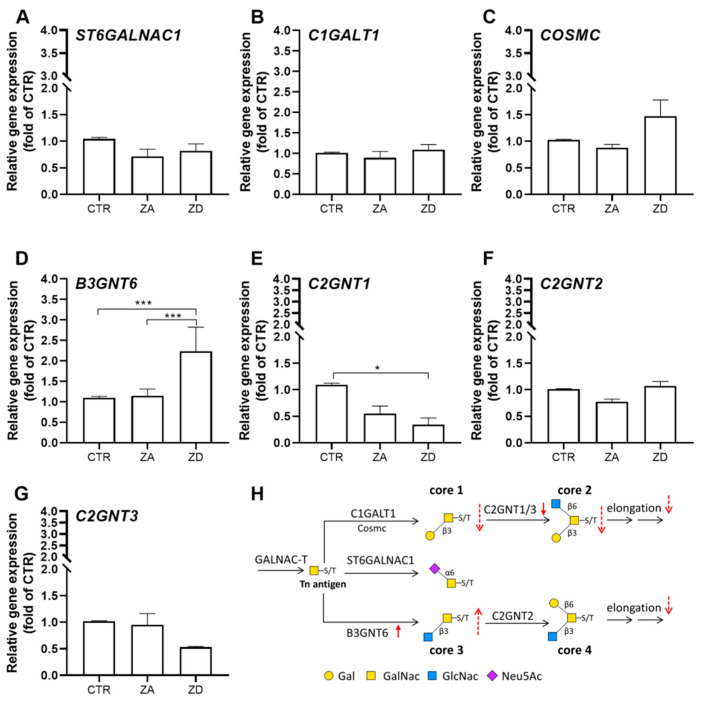
Impact of zinc deficiency on the expression of transferases responsible for the synthesis of core 1-4 *O*-glycans. (**A**–**G**) Expression of transferases that determine formation of the core structures 1-4 were analyzed using post-confluent HT-29-MTX cells, cultured in CTR, zinc-adequate (ZA) or -deficient (ZD) medium, respectively. (**H**) Initial steps of glycan biosynthesis, modified from [41], and changes of this process during zinc deficiency are shown (dashed arrows). Monosaccharides are depicted according to the symbol nomenclature for glycans (SNFG) [44]. Changes in gene expression of ST6 *N*-Acetyl-galactosaminide α-2,6-Sialyltransferase 1 (ST6GALNAC1), core 1 glycoprotein-*N*-acetylgalactosamine 3-β-galactosyltransferase (C1GALT1), the C1GALT1-specific molecular chaperone (COSMC), β-1,3-*N*-acetylglucosaminyl-transferase (B3GNT6) and β-1,6-*N*-acetylglucosaminyltransferase isoforms (C2GNT1, C2GNT2, C2GNT3) in zinc-deprived states are depicted with closed arrows (downward: downregulation; upward: upregulation). Data are presented as means + SEM of three independent experiments. Significant differences are indicated (* *p* < 0.05; *** *p* < 0.001; ANOVA with Bonferroni post hoc test).

ZD goblet cells produced higher amounts of mucins based on core 3 than on core 1 and particularly less core 2 glycans. This implies that zinc might not only influence the length of oligosaccharide chains but particularly elongation of the Tn antigen after the start of *O*-glycan biosynthesis. Mucus layer composition not only depends on the expression pattern of respective *MUC* genes but is primarily determined by the distribution and activity of glycosyltransferases [20]. Whether core 1 or core 3 mucins are produced mainly depends on the expression of three enzymes: core 1 glycoprotein-*N*-acetyl-galactosamine-3-β-galactosyl-transferase (C1GALT1), β-1,3-*N*-acetyl-glucosaminyltransferase (B3GNT6) and ST6GALNAC1 [45]. C1GALT1, whose function additionally relies on the presence of the C1GALT1-specific molecular chaperone (COSMC) [46], adds Gal in 1,3-linkage to GalNAc synthesizing core 1 glycans, whereas B3GNT6 catalyzes the formation of core 3 by adding GlcNAc [47]. ST6GALNAC1, on the other hand, terminates *O*-glycan elongation by adding sialic acid to Tn antigen [48] (Figure 7H). To this end, the impact of zinc deficiency on mRNA expression of the major core-producing enzymes was analyzed (Figure 7).

*ST6GALNAC1* mRNA levels were not affected by zinc deficiency (Figure 7A), which is in agreement with the comparable abundance of sialyl-Tn antigen detected in CTR and ZD cells (Figure 6A,B). While *C1GALT1* expression remained unaltered in ZD (Figure 7B), the mRNA of B*3GNT6*, essential for the synthesis of core-3, was significantly elevated (Figure 7D). Of note, the chaperone *COSMC*, essential for activity of the core 1 transferase, was slightly upregulated in ZD cells (Figure 7C). Zinc restriction seems to impact already the initial biosynthesis of core *O*-glycan structures, leading to a shift from core 1 to mainly core-3-based *O*-glycans, by influencing the transcription of the glycosyltransferases responsible for their formation. Similar changes in the *O*-glycan pattern were reported upon in vitro depletion of C1GALT1, which increased the formation of core 3 glycans as well as sialyl-Tn [45]. Additionally, C1GALT1 (−/−) knockout mice exhibit elevated levels of core 3 and 4; whereas B3GNT6 (−/−) mice produced more core 1 and core 2-based *O*-glycans [49]. Core 1 and core 3 are precursors of the subsequently formed two core structures. Consequently, the formation of core 2 and 4 also depend on the expression of C1GALT1 and B3GNT6, which might explain the diminished synthesis of core 2 by zinc-deprived cells (Figure 6). In the human GIT, three β-1,6-*N*-acetylglucosaminyltransferase (GNT) isoforms catalyze the addition of GlcNAc to the GalNAc residue of existing core 1 or 3 glycans, forming core 2 (isoforms C2GNT1 and C2GNT3) and core 4 (isoform C2GNT2), respectively [47]. In addition to the decline of precursor core 1, a decrease in core 2 *O*-glycans and overall elevated production of shorter *O*-glycans during zinc deficiency can partly be explained by diminished elongation of oligosaccharide-chains mediated by C2GNT1-3, as *C2GNT3* mRNA is slightly and *C2GNT1* is significantly downregulated (Figure 7E,G). To what extent protein abundance as well as activity of the initial glycosyltransferases are affected by zinc deficiency has to be further elucidated. Apart from the competing glycosyltransferases, the *O*-glycan composition is additionally depending on the availability of sugar-nucleotide donor substrate concentrations and their transport rate into the Golgi [50], the activity of specific chaperones [46] as well as the positioning of transferases in the Golgi [51]. The impact of zinc deficiency on these processes might also be worth investigating, as it may explain the impaired elongation of *O*-glycans and increased production of short sugar chains by ZD goblet cells.

Taken together, mucins produced by zinc-restricted goblet cells are not only changed in their MUC apo-protein expression but are mainly differentially glycosylated and consist of short *O*-glycans. These changes can affect the formation of the intestinal mucus layer as well as intestinal health. Glycosylation is crucial for the gel-forming ability of mucins along with the structure and stability of the intestinal mucus layer, which is already perturbed by slight differences of the *O*-glycan pattern [52]. Accordingly, the lack of zinc not only impairs the amount of secreted mucins but might also impact the stability of the mucus layer due to altered *O*-glycosylation. This also explains the deranged mucus formation in ZD animals and HT-29-MTX, visualized by histological staining. Consequently, the protective function of the intestinal mucus layer abates during zinc deficiency, leaving the underlying epithelium more vulnerable against the intestinal environment, including pathogens and commensal bacteria. This contributes to the overall degeneration of the intestinal mucosa observed during zinc deficiency [7,8,9] and increases the occurrence of intestinal infections, which promote diarrhea [53], a typical symptom of zinc deficiency [40]. Deterioration of this physical barrier could also disturb absorption of nutrients, as the mucus layer is known to be beneficial for nutrient absorption and discussed to enhance zinc absorption by improving its availability for the underlying epithelium [17]. The outcome of this study emphasizes the essentiality of zinc for mucus production, and thereby possibly also for intestinal health and immunity. Zinc might indirectly influence the GIT microbiome and intestinal diseases, such as inflammation, as it seems to modulate the *O*-glycan pattern of mucins, which is known to be important for host-microbiota interactions and intestinal homeostasis [19].

## 3. Materials and Methods

### 3.1. Materials

Alcian blue 8GX (Alfa Aesa, Karlsruhe, Germany), bicinchoninic acid (BCA) (Sigma Aldrich, Munich, Germany), Chelex^®^ 100 Resin (Bio-Rad, Hercules, CA, USA), 3-(4,5-Dimethylthiazol-2-yl)- 2,5-diphenyltetrazolium bromide (MTT) (Carl Roth, Karlsruhe, Germany), Dulbecco’s Modified Eagles Medium (DMEM) (PAN-Biotech, Aidenbach, Germany), Fetal calf serum (FCS) (CCPro, Oberdorla, Germany), iScript cDNA Synthesis Kit (Quantabio, Beverly, MA, USA), non-essential amino acids (NEAA) (Sigma Aldrich, Munich, Germany), NucleoSpin II (Macherey-Nagel GmbH & Co. KG, Berlin, Germany), pararosaniline hydrochloride (TCI, Eschborn, Germany), 100 U/mL penicillin and 100 µg/mL streptomycin (Sigma Aldrich, Munich, Germany), sulforhodamine B (SRB) (Sigma Aldrich, Germany), SYBR™-Green (Quantabio, Beverly, MA, USA), ZnSO_4_x7H_2_O (Sigma Aldrich, Munich, Germany). All other chemicals were purchased from standard sources.

### 3.2. Cells and Cell Culture

Goblet cells HT-29-MTX-E12 were obtained from the European Collection of Authenticated Cell Cultures (ECACC, Porton Down, UK). This cell line represents a well characterized subpopulation of the colon adenocarcinoma cell line HT-29, isolated after treatment with methotrexate to induce mucus production [54]. Cells were cultured in DMEM with phenol red, including 10% FCS and 100 U/mL penicillin and 100 µg/mL streptomycin, and 1% NEAA and at 37 °C with 5% CO_2_ until confluency. Depending on the experiment, cells (initial cell number: 96 well plate: 10,000 cells per well; 6-well plate: 250,000 cells per well) were cultured for 4–14 days, medium was changed every other day.

### 3.3. Preparation of Cell Culture Medium

ZD medium was obtained by incubating complete medium (CTR; DMEM with phenol red, containing 10% FCS, 100 U/mL penicillin, 100 µg/mL streptomycin, and 1% NEAA) with Chelex^®^ 100 Resin (50 g/L medium), a styrene divinylbenzene copolymer containing paired iminodiacetate ions, for 24 h (zinc content of complete medium: 2.8 ± 0.06 µM, zinc content of ZD medium: <limit of quantitation (LOQ) (Supplementary Table S3; conditions for quantitation of metals (Zn, Cu, Mn) via inductively coupled mass spectrometry (ICP–MS) are summarized in Supplementary Table S4 and via flame atomic absorption spectrometry (FAAS) (Ca, Mg) in Supplementary Table S5). Calcium, magnesium, copper and manganese, which were also removed during Chelex^®^-treatment (Supplementary Table S3), were replenished and chelexed medium was sterile filtered (0.2 µm cut off filter, Sigma Aldrich, Munich, Germany). Zinc-adequate (ZA) medium was prepared by adding the amount of removed zinc back into ZD medium.

### 3.4. Cellular Protein Content

Cells in 96-well plates were cultured with CTR, ZD and ZA medium for 4, 7, 11 and 14 days and cellular protein was determined by SRB assay as described [55].

### 3.5. Cell Viability

After culturing cells with ZD and ZA medium in 96-well plates for 7 or 14 days, cells were incubated with 0–1000 µM ZnSO_4_·7H_2_O in DMEM w/o phenol red and 0% FCS for 24 h. Subsequently, the dehydrogenase activity of cells was analyzed by MTT assay as described [55].

### 3.6. Cellular Zinc Uptake

Cells cultured in CTR, ZA or ZD medium for 7 or 14 days were incubated with 0–100 µM ZnSO_4_·7H_2_O in DMEM w/o phenol red and 0% FCS for 24 h. Cells were harvested with phosphate buffered saline on ice, an aliquot was collected for protein quantification using BCA assay [56], and cellular zinc was determined by ICP–MS as reported [17].

### 3.7. Gene Expression

Cells were cultured in 6-well plates for 7 or 14 days and harvested on ice. RNA was isolated with the Nucleo Spin II Kit, cDNA was synthesized with the iScript cDNA Synthesis Kit and mRNA-levels were quantified by quantitative real-time PCR (qPCR) with SYBR™Green Super Mix on an iCycler Optical System (Bio-Rad Laboratories, Hercules, CA, USA), using primers and thermal cycling conditions listed in Supplementary Tables S6 and S7. Relative quantification of mRNA was realized using the 2^δδCt^-method [57] with Ct-values normalized to β-ACTIN and referred to cells cultured with CTR medium for 7 days.

### 3.8. Histological Staining of Mucins

Cells were cultivated in 6-well plates on glass coverslips for 14 days and secreted mucus of HT-29-MTX was visualized by histological staining with AB and PAS as reported [55]. Images were acquired using an Axio Imager M1 microscope equipped with an Axiocam 503 mono and processed with Zen 2.3 software (hardware and software from Carl Zeiss Microscopy, Jena, Germany).

### 3.9. Analysis of O-glycosylation of Secreted Mucins by MALDI-TOF MS

HT-29-MTX were cultured for 14 days in 150 cm^2^ dishes (initial cell number: 2 × 10^6^ cells/dish) with CTR or ZD medium. Twenty-four hours before collecting secreted mucins, medium was changed to DMEM w/o phenol red and 0% FCS. Supernatants were purified and oligosaccharides were released from mucins by alkaline borohydride treatment as described [42]. After the permethylation of oligosaccharides, the *O*-glycosylation of mucins was analyzed by matrix-assisted laser desorption/ionization time-of-flight (MALDI-TOF) mass spectrometry in the positive ion mode [42].

### 3.10. Statistical Analysis

Statistical significance was analyzed by one- or two-way analysis of variance (ANOVA), followed by Bonferroni or Dunnett’s multiple comparison post hoc tests, as indicated in the respective figure legends, using GraphPad Prism software version 8 (GraphPad Software Inc., San Diego, CA, USA). Error bars represent the standard deviation (SD) or standard error of mean (SEM) of three independent biological replicates.

## 4. Conclusions

This study demonstrates the essentiality of nutritional zinc for cell differentiation and mucus production of intestinal goblet cells. Similar to what is known for enterocytes, the zinc homeostasis of goblet cells during zinc deficiency is regulated by differential expression of zinc transporters to counterbalance the differing nutritional zinc availability. A lack of this essential metal significantly upregulated *MUC2* and severely impaired the secreted and gel-forming mucus layer. Degeneration and the disturbed stability of mucus during zinc deficiency seem to be mostly caused by a perturbed mucin synthesis on the post-translational level, leading to an altered *O*-glycosylation pattern. The outcome of this examination underlines the importance of the initial glycosyltransferases in this alteration, being responsible for initial *O*-glycan biosynthesis and leading to a shift of core structures and the production of shorter *O*-glycans during zinc deficiency. Consequently, observed changes in the *O*-glycan pattern of the intestinal mucus layer along with extremely reduced mucin secretion during zinc deficiency explains the disruption of this physical barrier and the impairment of intestinal health during this nutrient’s deficiency.

## Supplementary Materials

Supplementary materials can be found at https://www.mdpi.com/1422-0067/21/17/6149/s1.

## Abbreviations

AB	Alcian blue
ANOVA	Analysis of variance
BCA	Bicinchoninic acid
B3GNT6	β-1,3-*N*-acetylglucosaminyltransferase
C1GALT1	Core 1 glycoprotein-*N*-acetylgalactosamine 3-β-galactosyltransferase
CF	Cystic fibrosis
COSMC	C1GALT1-specific molecular chaperone
DMEM	Dulbecco’s Modified Eagles Medium
ECACC	European Collection of Authenticated Cell Culture
FAAS	Flame atomic absorption spectrometry
FCS	Fetal calf serum
GalNAc	*N*-acetyl-galactosamine
GIT	Gastrointestinal tract
GNT	β-1,6-*N*-acetylglucosaminyltransferase
MALDI-TOF	Matrix-assisted laser desorption/ionization time-of-flight
NeuAc	*N*-acetylneuraminic acid
ICP–MS	Inductively coupled plasma mass spectrometry
LOQ	Limit of quantitation
NEAA	Non-essential amino acids
PAS	Periodic acid Schiff
GalNAc-Ts	UDP-GalNAc:polypeptide GalNAc transferases
ST6GALNAC1	ST6 *N*-Acetylgalactosaminide Alpha-2,6-Sialyltransferase 1
TPEN	*N*,*N*,*N*′,*N*′-Tetrakis(2-pyridylmethyl)ethylenediamine
ZA	Zinc-adequate
ZD	Zinc-deficient
ZnT	Zinc transporter
ZIP	Zrt Irt-like transporter
