# Supplementary material for: Zinc Deficiency Disturbs Mucin Expression, O-Glycosylation and Secretion by Intestinal Goblet Cells
Source: Int J Mol Sci. 2020 Aug 26;21(17):6149. doi: 10.3390/ijms21176149 (PMC7504335; doi:10.3390/ijms21176149)
Supplement: Supplementary file 1 [file ijms-21-06149-s001.pdf]

Zinc deficiency disturbs mucin expression, O-glycosylation and secretion by intestinal goblet cells

Maria Maares [1,*], Claudia Keil [1], Sophia Straubing [1], Catherine Robbe-Masselot [2], Hajo Haase [1,3]

[1] Technische Universität Berlin, Chair of Food Chemistry and Toxicology, Straße des 17. Juni 135, 10623 Berlin, Germany

[2] Univ. Lille, CNRS, UMR8576-UGSF-Unité de Glycobiologie Structurale et Fonctionnelle, Lille, France

[3] TraceAge - DFG Research Unit on Interactions of essential trace elements in healthy and diseased elderly, Potsdam-Berlin-Jena, Germany

*Corresponding author: Technische Universität Berlin, Chair of Food Chemistry and Toxicology, Straße des 17. Juni 135, 10623 Berlin, Germany

Tel.: +49 (0) 30 31472816

Fax: +49 (0) 30 31472823

E-Mail: Maares@TU-Berlin.de

	HT-29-MTX (7 d)		HT-29-MTX (14 d)	
	ZD	ZA	ZD	ZA
**Best-fit values**				
Bottom	-4.154	5.183	~ -1657	~ -425.2
Top	104.4	94.21	87.07	96.00
Hill slope	-1.707	-1.569	~ -4.037	~ -5.052
LC_{50}	259.1	349.8	~ 2119	~ 1401
95% Confidence Interval of LC_{50}	214 to 334.3	262.9 to 1284	Very wide	Very wide
Goodness of Fit	38	38	38	38
Degree of Freedom	0.9634	0.9372	0.7223	0.7668
R^2	2105	2017	12490	9967
Absolute Sum of Squares	7.443	7.286	18.13	16.20

Supplementary Table S1: Parameters of the non-linear regression analysis applied in the zinc cytotoxicity study in Fig. 2. Shown are parameters of the applied non-linear regression using a sigmoidal dose-response curve with variable slope as a function of the logarithm of zinc concentration. Data were obtained in three independent experiments and analyzed with GraphPad Prism software version 8 (GraphPad Software Inc., San Diego, CA, USA).

Supplementary Table S2: Structure of O-glycans found in secreted mucins of HT-29-MTX

m/z	Core structure	O-glycan	structure
534	Core 1	Galβ1-3GalNAc (TF antigen)	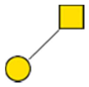
575	Core 3	GlcNAcβ1-3GalNAc	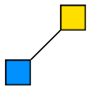
663	Core 3	sulfated GlcNAcβ1-3GalNAc	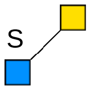
691		NeuAcα2-6GalNAc (sialyl Tn antigen)	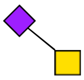
779	Mix of core 1-3	core 1:GlcNAcβ1-3Galβ1-3GalNAc, core 2:Galβ1-3(GlcNAcβ1-6)GalNAc and core 3:Galβ1-3GlcNAcβ1-3GalNAc	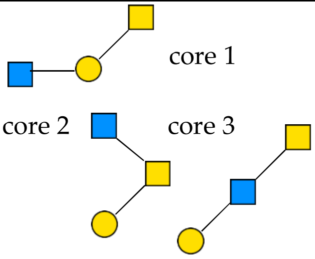
867	Mix of core 1-3	mix of sulfated core 1, core 2 and core 3	
895	Core 1	NeuAcα2-3Galβ1-3GalNAc **or** Galβ1-3(NeuAcα2-6)GalNAc (sialyl TF antigen)	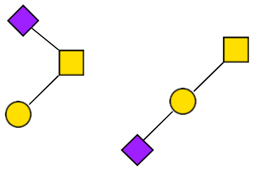
1069	Core 1	Fucα1-2Galβ1-3(NeuAcα2-6)GalNAc	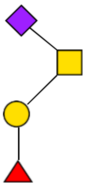
1140	Core 2 or core 3	NeuAcα2-3Galβ1-3/4GlcNAcβ1-3GalNAc (core 3, higher abundance) or NeuAcα2-3Galβ1-3(GlcNAcβ1-6)GalNAc (core 2)	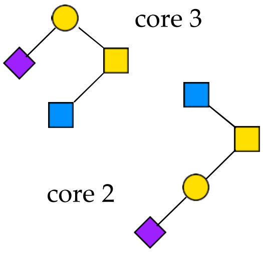

1256	Core 1	NeuAcα2-3Galβ1-3(NeuAcα2-6)GalNAc (disialylated TF antigen)	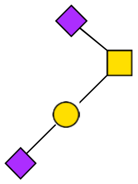
1677		probably core 2	
1705	Core 2	NeuAcα2-3Galβ1-3(NeuAcα2-3Galβ1-4GlcNAcβ1-6)GalNAc	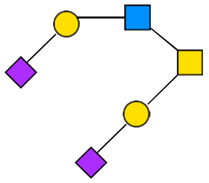

Monosaccharides are depicted according to symbol nomenclature for glycans (SNFG) [1].

Supplementary Table S3: Metal content after treatment with Chelex® 100 Resin

Medium	Zn (μg/L) *	Cu (μg/L) *	Mn (μg/L) *	Ca (mg/L)**	Mg (mg/L)**
Complete DMEM	182.7±4.1	23.1±0.5	5.1±0.1	82.8±10.9	19.4±0.0
Chelexed DMEM	<LOQ	13±0.3	3.7±0.2	0.2±0.001	<LOQ

*quantified by inductively-coupled plasma mass spectrometry (ICP-MS)
**quantified by flame atomic absorption spectrometry (FAAS)

Supplementary Table S4: Experimental conditions for ICP-MS (Perkin Elmer ELAN DRC 600)

Forward power	1550 W
Cool gas flow	15 L min^{-1}
Auxiliary gas flow	0.9 L min^{-1} (Argon)
Nebulizer gas flow	0.9 L min^{-1} (Argon)
Nebulizer type	MicroMist
Quadrupole (m/z)	66 (Zn), 55 (Mn), 63 (Cu),111 (Cd), 103 (Rh)
Limit of quantitation	0.2 μg L^{-1} (Zn); 0.1 μg L^{-1}(Mn); 0.5 μg L^{-1} (Cu); 0.15 μg L^{-1} (Cd)
Calibration range	1-100 μg L^{-1} (Zn, Mn, Cu); 0.01-1 μg L^{-1} (Cd)

Supplementary Table S5: Experimental conditions for FAAS (Perkin Elmer AAnalyst 800)

Gas flow	Acetylen 2.0 L min^{-1}<br>Oxygen 17 L min^{-1}
Lamp	Hollow Cathode Lamp
Wavelength [nm]	422.7 nm (Ca); 285.2 nm (Mg)
Slit [nm]	0.7 nm
Lamp Current	6 mA
Limit of quantitation	0.12 mg L^{-1} (Ca); 0.02 mg L^{-1} (Mg)
Calibration range	0.1-5 mg L^{-1} (Ca); 0.01-0.5 mg L^{-1} (Mg)

Supplementary Table S6: Oligonucleotide sequences used for real-time PCR

Primer	NCBI Reference Sequence	Sequence fwd 5'-3'	Sequence rev 5'-3'	Product size (bp)	Assay performance[a]		T (Annealing °C)	Ref
					PCR Efficiency[a]	R^2		
ZIP-4	NM_017767	AGACTGAGCCCAGAGTTGAGGCTA	TGTCGCAGAGTGCTACGTAGAGGA	352	1.0	0.9937	58	[2]
ZIP-5	NM_173596	GAGCAGGAGCAGAACCATTACCTG	CAATGAGTGGTCCAGCAACAGAAG	354	0.8	0.9621	58	[2]
ZnT1	NM_021194	GGCCAATACCAGCAACTCCAA	TGCAGAAAAACTCCACGCATGT	175	1.1	0.9896	58	[3]
ZnT5B	NM_024055	AAGGACATCATGACAGTGCTCTAACTC	CCAACTTTACAACACAAAGCCAGTAC	118	1.0	0.9907	58	[3]
MUC2	NM_002457.4	CAGCTCATCTCGTCCGTCTC	GCTGGCTGGTTTTCTCCTCT	298	1.3	0.972	60	[4]
MUC5AC	NM_001304359.1	CATCAACGGGACCCTGTACC	ACAGGTCGACTGGTTCTGGT	445	1.0	0.9926	60	[5]
C1GALT1	NM_020156.5	TGGGAGAAAAGGTTGACACC	CGGTCATAACCCAGCAAAGA	195	1.8	0.9613	58	
B3GNT6	NM_138706.5	TCAACCTCACGCTCAAGCAC	CAGGAAGCGGACTACGTTGG	125	1.3	0.9938	58	[6]
COSMC	NM_001011551.3	GCCAACGTGAGAGGAAACC	GCTCATGGTGGTGCATTCTA	190	2.1	0.9791	58	
C2GNT1	NM_001097633.1	CGCACACATTTTCAACAACC	GCAGTCTGGGAAGACTGAGG	183	2.3	0.916	58	
C2GNT2	NM_001374747.1	TGTTCCTGGCTCTATGCCAAA	TTAGCAAACAGGCTTGGTGAAT	171	9.5	0.8802	58	[7]

C2GNT3	NM_004751.3	GCTTCCCGAGATTTCGTCCA	AACAGAGCCAGGCATCCACC	138	4.0	0.9258	58	[6]
ST6GALNAC1	NM_001289107.2	GGACTATGAGTGGCTGGAAGCA	CTGGTACAGCCGGATTATCCCT	421	1.1	0.9883	60	[8]
β-ACTIN	NG_007992.1	CGCCCCAGGCACCAGGGC	GCTGGGGTGTTGAAGGT	284	0.8		58 or 60	[9]

bp, base pair; [a] Assay performance was determined according to the MIQE (Minimum Information required for publication of Q-PCR Experiments) guidelines [10]

Supplementary Table S7: Thermal cycling conditions of real-time PCR

Cycles		Time	Temperature (°C)
1	Initial hot start	1.5 min	95
40	Denaturation	30 sec	95
	Annealing	30 sec	refer to Supplementary Table 4
	Extension	45 sec	72
1	Dissociation curve	1 min	95
1		1 min	60
70		1 min	55-60 °C (in 0.5°C increments)
hold	End	∞	4

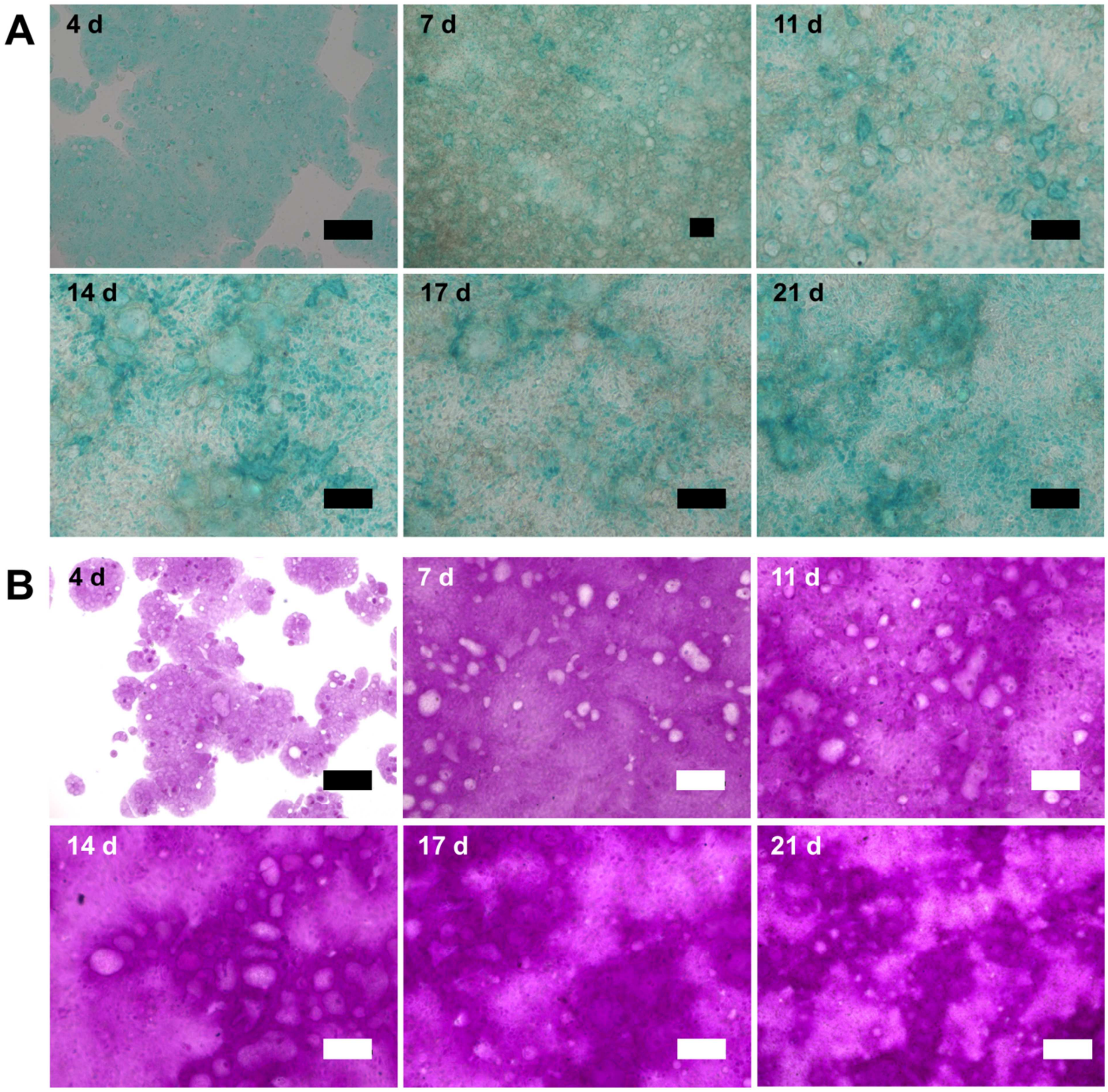

Supplementary Figure S1: Mucin secretion of HT-29-MTX during cell growth and differentiation. Extracellular mucins of zinc-sufficient pre-confluent (4 d), confluent (7 d, 11 d) and post-confluent (14 d – 21 d) HT-29-MTX cells are visualized with histological staining: Acidic mucins were stained with alcian blue (A) and neutral mucins with periodic acid Schiff (PAS)-assay (B). Images were taken with a digital microscope from Keyence (Germany). Scale bar 100 μm.

References

1. Varki, A.; Cummings, R.D.; Aebi, M.; Packer, N.H.; Seeberger, P.H.; Esko, J.D.; Stanley, P.; Hart, G.; Darvill, A.; Kinoshita, T.*, et al.* Symbol nomenclature for graphical representations of glycans. *Glycobiology* **2015**, *25*, 1323-1324.
2. Leung, K.W.; Liu, M.; Xu, X.; Seiler, M.J.; Barnstable, C.J.; Tombran-Tink, J. Expression of znt and zip zinc transporters in the human rpe and their regulation by neurotrophic factors. *Investigative Ophthalmology & Visual Science* **2008**, *49*, 1221-1231.
3. Overbeck, S.; Uciechowski, P.; Ackland, M.L.; Ford, D.; Rink, L. Intracellular zinc homeostasis in leukocyte subsets is regulated by different expression of zinc exporters znt-1 to znt-9. *Journal of Leukocyte Biology* **2008**, *83*, 368-380.
4. Gouyer, V.; Wiede, A.; Buisine, M.P.; Dekeyser, S.; Moreau, O.; Lesuffleur, T.; Hoffmann, W.; Huet, G. Specific secretion of gel-forming mucins and tff peptides in ht-29 cells of mucin-secreting phenotype. *Biochimica et biophysica acta* **2001**, *1539*, 71-84.
5. Maares, M.; Duman, A.; Keil, C.; Schwerdtle, T.; Haase, H. The impact of apical and basolateral albumin on intestinal zinc resorption in the caco-2/ht-29-mtx co-culture model. *Metallomics* **2018**, *10*, 979-991.
6. Ye, J.; Wei, X.; Shang, Y.; Pan, Q.; Yang, M.; Tian, Y.; He, Y.; Peng, Z.; Chen, L.; Chen, W.*, et al.* Core 3 mucin-type o-glycan restoration in colorectal cancer cells promotes muc1/p53/mir-200c-dependent epithelial identity. *Oncogene* **2017**, *36*, 6391-6407.
7. Peng, F.; He, Q.; Cheng, C.; Pan, J. Gcnt2 induces epithelial-mesenchymal transition and promotes migration and invasion in esophageal squamous cell carcinoma cells. *Cell Biochemistry and Function* **2019**, *37*, 42-51.
8. Julien, S.; Krzewinski-Recchi, M.A.; Harduin-Lepers, A.; Gouyer, V.; Huet, G.; Le Bourhis, X.; Delannoy, P. Expression of sialyl-tn antigen in breast cancer cells transfected with the human cmp-neu5ac: Galnac alpha2,6-sialyltransferase (st6galnac i) cdna. *Glycoconj J* **2001**, *18*, 883-893.
9. Wolf, K.; Schulz, C.; Riegger, G.A.J.; Pfeifer, M. Tumour necrosis factor-α induced cd70 and interleukin-7r mrna expression in beas-2b cells. *European Respiratory Journal* **2002**, *20*, 369-375.
10. Bustin, S.A.; Benes, V.; Garson, J.A.; Hellemans, J.; Huggett, J.; Kubista, M.; Mueller, R.; Nolan, T.; Pfaffl, M.W.; Shipley, G.L.*, et al.* The miqe guidelines: Minimum information for publication of quantitative real-time pcr experiments. *Clinical Chemistry* **2009**, *55*, 611-622.
